# Theory Meets Experiment: Metal Ion Effects in HCV Genomic RNA Kissing Complex Formation

**DOI:** 10.3389/fmolb.2017.00092

**Published:** 2017-12-22

**Authors:** Li-Zhen Sun, Xiao Heng, Shi-Jie Chen

**Affiliations:** ^1^Department of Applied Physics, Zhejiang University of Technology, Hangzhou, China; ^2^Department of Physics, University of Missouri, Columbia, MO, United States; ^3^Department of Biochemistry, University of Missouri, Columbia, MO, United States; ^4^University of Missouri Informatics Institute, University of Missouri, Columbia, MO, United States

**Keywords:** HCV, RNA folding, metal ion effects, PCTBI model, Vfold model, NMR

## Abstract

The long-range base pairing between the 5BSL3. 2 and 3′X domains in hepatitis C virus (HCV) genomic RNA is essential for viral replication. Experimental evidence points to the critical role of metal ions, especially Mg^2+^ ions, in the formation of the 5BSL3.2:3′X kissing complex. Furthermore, NMR studies suggested an important ion-dependent conformational switch in the kissing process. However, for a long time, mechanistic understanding of the ion effects for the process has been unclear. Recently, computational modeling based on the Vfold RNA folding model and the partial charge-based tightly bound ion (PCTBI) model, in combination with the NMR data, revealed novel physical insights into the role of metal ions in the 5BSL3.2-3′X system. The use of the PCTBI model, which accounts for the ion correlation and fluctuation, gives reliable predictions for the ion-dependent electrostatic free energy landscape and ion-induced population shift of the 5BSL3.2:3′X kissing complex. Furthermore, the predicted ion binding sites offer insights about how ion-RNA interactions shift the conformational equilibrium. The integrated theory-experiment study shows that Mg^2+^ ions may be essential for HCV viral replication. Moreover, the observed Mg^2+^-dependent conformational equilibrium may be an adaptive property of the HCV genomic RNA such that the equilibrium is optimized to the intracellular Mg^2+^ concentration in liver cells for efficient viral replication.

## Introduction

Belonging to the *Flaviviridae* family, hepatitis C virus (HCV) is the only member of the *Hepacivirus* genus. It has infected nearly 200 million individuals around the globe. The viral genome is a 9.6 kb single-stranded, positive-sense RNA, which not only encodes viral proteins but also encrypts regulation of viral replication steps (Moradpour et al., 2007). The 5′-untranslated region (UTR) contains a highly structured internal ribosomal entry site (IRES) that initiates translation of the polyprotein encoded in the open reading frame (ORF). The polyprotein translated from the single ORF is processed by host and viral proteases to produce structural proteins and nonstructural proteins. Minus-strand RNA synthesis initiates at the 3′-UTR by viral nonstructural protein 5B (NS5B), which also produces positive-strand RNA using the minus-strand RNA as template (Moradpour et al., 2007).

Both viral protein translation and minus-strand RNA synthesis occur on the genomic RNA (gRNA). Hence, mechanism must exist to prevent collision of these two replication machineries. While viral and host proteins contribute significantly to the regulation, dynamic RNA structures and RNA:RNA interactions in the gRNA also play a pivotal role. Current knowledge has posited the 3′-UTR of the gRNA as the key regulatory element. It serves as a hub participating in long-range interactions with the 5BSL3.2 stem loop in the ORF (You et al., 2004; Friebe et al., 2005; You and Rice, 2008), as well as the 5′- internal ribosome entry site (IRES) (Romero-López and Berzal-Herranz, 2009; Romero-Lopez et al., 2012; Romero-López et al., 2014). The base pair complementarities between 5BSL3.2 and the 3′-tail of the gRNA (3′X, 98 nucleotides), are critical for viral replication (You et al., 2004; Friebe et al., 2005; You and Rice, 2008), and nucleotide accessibility probing of the 3′-UTR has demonstrated its structural changes in the presence of upstream elements (Romero-Lopez et al., 2012; Romero-López et al., 2014; Tuplin et al., 2012). However, lack of accurate models of the RNA:RNA interaction network has hampered functional and mechanistic interpretations. One of the main challenges for studying RNA:RNA interactions is about how to understand and predict the effects of metal ions, which are essential for neutralizing/screening RNA backbone charges (Brion and Westhof, 1997; Tinoco and Bustamante, 1999; Davis et al., 2007; Fürtig et al., 2010).

## Computational modeling of ion-RNA interactions

Ions bound to an RNA structure can be classified into three types (Cate and Doudna, 1996; Draper et al., 2005; Tan and Chen, 2005): site-specific bound (SSB) ions (Cate and Doudna, 1996), diffusively bound (DB) ions (Draper et al., 2005), and tightly bound (TB) ions (Tan and Chen, 2005). SSB ions are fully or partially dehydrated and trapped at specific sites such as the pocket regions in the RNA structure (Misra and Draper, 2001). Because of the chelation with the electronegative atomic groups of RNA, SSB ions have relatively specific binding sites in the RNA (Ando et al., 2003; Lilley, 2003; Forconi and Herschlag, 2009; Kellerman et al., 2014; Bobyr et al., 2015). DB ions are fully hydrated and bound to RNA diffusively (Draper et al., 2005). These ions can diffuse at a distance from the surface of RNA. TB ions are clustered around RNA surface to form a high local concentration (Tan and Chen, 2005). TB ions form a mid-state between SSB and DB ions (Sun et al., 2017a): on the one hand, TB ions do not involve SSB-like specific binding sites and are hydrated, on the other hand, TB ions cannot move around like DB ions, because the local high concentration and the resultant ion-ion coupling (correlation) can significantly lower the ion mobility. The ion correlation prevents a fluid-like model mean-field treatment for the TB ions. The TB ions should be treated as explicit, discrete particles.

Molecular dynamic (MD) simulations can account for the ion correlation effect since the ions are treated as explicit particles (Joung and Cheatham, 2009; Kuczera et al., 2009; Hayes et al., 2014). However, the simulations are limited by the computational time (Dong et al., 2008). Mean-field based non-simulation models, such as the non-linear Poisson-Boltzmann (NLPB) model (Zhou, 1994; Misra and Draper, 1998, 1999; Luo et al., 2002; Claudia et al., 2007) and the counterion condensation (CC) theory (Manning, 1978) cannot treat ion correlations. As a result, although NLPB has been highly successful in predicting the electrostatic effects for (weakly correlated) monovalent ions, the theory may under-estimate ion-mediated stabilization effect for (strongly correlated) multivalent ions such as Mg^2+^ ions (Bai et al., 2007; Gebala et al., 2015). With the increasing recognition of the importance of ion correlation effect, several new models have been developed (Mak and Henke, 2013; Henke and Mak, 2014), such as three-dimensional reference interaction site model (3D-RISM) (Giambaşu et al., 2014, 2015), which can simultaneously account for the ion correlation and solvation (dehydration/hydration) effects, and the generalized counterion condensation theory (Hayes et al., 2015), which adds a Mg^2+^-Mg^2+^ interaction term to the traditional CC theory. These models have shown promising potential in the prediction of the ion binding effects (Sun et al., 2017b).

The tightly bound ion (TBI) model (Tan and Chen, 2005) is one of the reliable models for the prediction of the ion effects in RNA folding. Extensive theory-experiment comparisons have shown that TBI model may provide improved predictions (Tan and Chen, 2010; He and Chen, 2012). The key feature of the model is to sample discrete multi-ion distributions for the TB ions and for each distribution, to evaluate the multi-ion correlated electrostatic free energy for the system (RNA and the ion solution). Through the use of the Generalized Born model, the TBI model can quantify the different energetics components, such as the solvent polarization effects in ion-RNA interactions (He and Chen, 2013). The ability to treat ion correlation and fluctuation, which can be important for multivalent ions such as Mg^2+^, makes TBI an ideal model for the HCV gRNA folding system, which involves Mg^2+^ ions.

Since the original TBI framework was first reported, several new models have been developed in the TBI framework. Most recently, using a novel sampling-resampling algorithm, the Monte Carlo tightly bound ion model (MCTBI) (Sun and Chen, 2016) was developed in order to enhance the computational efficiency, and the partial charge-based tightly bound ion model (PCTBI) (Sun et al., 2017a) was developed to account for more detailed RNA charge distribution. These new models have led to 1,000-fold improvement in computational time and thus allow us to treat large RNAs (several hundred nucleotides) (Sun et al., 2017c). More importantly, these new models enable accurate predictions for RNA-RNA interactions, such the 5BSL3.2:3′X kissing interactions in HCV gRNA below, where high-resolution charge distribution can be very important. However, the above models do not account for the heterogeneity of RNA conformations. Thus, in order to predict RNA structural changes, we need to sample RNA conformations using RNA folding models such as Vfold (Xu et al., 2014) and molecular dynamics (MD) simulations, and compute the ion-induced free energy changes for the different conformations. In what follows, we focus on the major conclusions and the analysis for the ion binding effects in the 5BSL3.2:3′X HCV kissing complex formation (Kranawetter et al., 2017; Sun et al., 2017a).

Since the demonstration of the importance of sequence complementarity of 5BSL3.2 and 3′X, efforts have been made to investigate the long-range kissing interaction at the 3′-end of gRNA. Starting from the 2D structure (base pair assignments) derived from the NMR experiment, the Vfold RNA folding model (Xu et al., 2014) gives the initial 3D structures. Starting from the initial 3D structures, MD simulation generates ensembles of 3D conformations. In the simulations, the base pairs in the initial structures are constrained by a harmonic potential $U\left(r\right)=k{\left(r-r_{0}\right)}^{2}$ with *k* = 1.0 kcal/mol and *r*_0_ = 3.5 Å. For each 3D conformation, the use of the PCTBI model gives the electrostatic free energy for the RNA-ion system:

$$\begin{array}{r} \Delta {\text{G}}_{3\text{D}}=\Delta {\text{G}}_{\text{ion}}+\Delta {\text{G}}_{\text{RNA}}, \end{array}$$

where ΔG_ion_ is the electrostatic free energy induced by the ions (including the TB and DB ions) and ΔG_RNA_ is the mutual electrostatic interaction between RNA backbone charges. The Boltzmann-weighted free energy ΔG = < ΔG_3D_ > describes the energy landscape of the system for the given ion condition.

## Mg^2+^ ion effects in the formation of the 5BSL3.2:3′X kissing complex

### 5BSL3.2 structures

**Figure 1:**
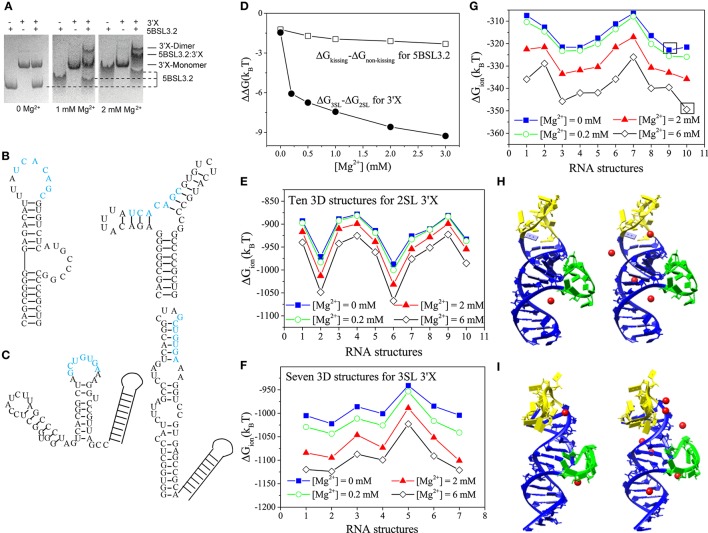
**(A)** 5BSL3.2 and 3′X mixtures were analyzed by native polyacrylamide gels containing 0, 1, and 2 mM [Mg^2+^] (left, middle, and right, respectively). **(B,C)** The kissing (left) and the three-way junction non-kissing (right) structures for 5BSL3.2 **(B)** and the three-stem loop (3SL; left) and the two-stem loop (2SL; right) structures for 3′X **(C)**. **(D)** Electrostatic free energy change with the increase in [Mg^2+^]. **(E–G)** The ion electrostatic free energies for the different 3D conformations of 2SL 3′X **(E)**, 3SL 3′X **(F)**, and 5BSL3.2 **(G)** at the different [Mg^2+^]'s, respectively. **(H,I)** The structures #9 **(H)** and #10 **(I)** with the predicted bound Mg^2+^ ions at [Mg^2+^] = 0.2 mM (left) and 2 mM (right). The hairpin and the bulge loops are labeled by yellow and green, respectively. All the solutions contain 150 mM KCl buffer.

For the 5BSL3.2 segment, NMR studies revealed that its secondary structure is comprised of a bottom base paired stem, a bulge and an upper single stranded region containing the SL2-complementary sequence (Friebe et al., 2005; Kranawetter et al., 2017; Sun et al., 2017a), as shown in Figure 1C (kissing 5BSL3.2). The Vfold model predicts, in addition to the above kissing conformation with the SL2-complementary sequence exposed, an alternative three-way-junction (non-kissing) conformation (the “non-kissing conformation” in Figure 1C). The non-kissing conformation of 5BSL3.2 is not compatible with the kissing complex with 3′X, because the SL2-complementary sequence (labeled in blue) is protected in the base paired stems.

### 3′X structures

In light of its essential role in viral replication, the structure of the 98-nucleotide 3′X has been investigated by enzymatic and chemical probing (Blight and Rice, 1997; Romero-López and Berzal-Herranz, 2012; Palau et al., 2013; Shetty et al., 2013). A three-hairpin (stem-loop) (3SL; see Figure 1B) model has been proposed as the kissing-loop residues in SL2 are exposed for interacting with 5BSL3.2. However, recent NMR and SAXS studies from two independent groups provide consistent evidence supporting a two-hairpin (2SL; also see Figure 1B) structural model (Cantero-Camacho and Gallego, 2015; Cantero-Camacho et al., 2017; Kranawetter et al., 2017). The kissing-loop residues (labeled by blue in Figure 1B) are sequestered in the extended SL2/3 stem loop structure, even in the context of full 3′-end under low ionic buffer conditions (Kranawetter et al., 2017).

### Ion-RNA interactions for the 5BSL3.2-3′X system

Native polyacrylamide gel electrophoresis experiment shows the following ion effects for the 5BSL3.2:3′X kissing complex system (Kranawetter et al., 2017; Sun et al., 2017a). For the 5BSL3.2 domain, gel shifting assays (first, forth, and seventh lanes in Figure 1A) show that the migration rate of the 5BSL3.2 segment depends on the Mg^2+^ concentration, suggesting that the 5BSL3.2 folding is sensitive to the presence of Mg^2+^ ion. In the follow-up NMR experiment, new imino signals (assigned to the residues of the 5BSL3.2 upper stem) were detected at higher [Mg^2+^], indicating a Mg^2+^-induced structural change in the 5BSL3.2 domain. In contrast, the folding of the 3′X RNA (second, fifth, and eighth lanes in Figure 1A), unlike 5BSL3.2, is much less sensitive to the presence of Mg^2+^ ion.

Furthermore, experiments show that RNA oligonucleotides of 5BSL3.2 and 3′X require Mg^2+^ to form a complex under *in vitro* conditions (Shetty et al., 2013; Cantero-Camacho and Gallego, 2015). In the experiments, the 5BSL3.2:3′X complex was loaded to native polyacrylamide gels with 0, 1, and 2 mM [Mg^2+^]. Results (see the third, sixth, and ninth lanes in Figure 1A) show that the 5BSL3.2:3′X complex can only be detected with the presence of sufficient Mg^2+^ ions, indicating that the long-distance RNA:RNA interactions at the 3′-end of gRNA are Mg^2+^-dependent. Therefore, the formation of the 5BSL3.2:3′X kissing complex requires Mg^2+^ ion, which is necessary to prevent the dissociation of the 5BSL3.2:3′X complex.

To understand how the metal ions shift the equilibrium between the alternative conformers requires an integrated theory-experiment approach. For each RNA (5BSL3.2, 3′X, and the 5BSL3.2:3′X kissing complex), the use of the Vfold/MD method can generate respective 3D conformational ensembles, while the use of the PCTBI model can predict the electrostatic free energies for the different ionic solution conditions. The free energy landscape, predicted as a function of the ion concentration, shows how ions shift the conformational distribution and induce conformational switch. The NMR data provides the 2D constraints (base pairs) for structure predictions. Furthermore, NMR and gel electrophoresis experiments give direct evidence for the ion effects which can be predicted by the theory.

### Ion effects for 5BSL3.2 and 3′X

The Vfold/MD method generates respective 3D conformational ensembles for the 2SL and 3SL 3′X folds, and the PCTBI model predicts the electrostatic free energy difference ΔΔG = ΔG_3SL_-ΔG_2SL_ between the two structures (see Figure 1D). The decrease of ΔΔG with an increasing [Mg^2+^] shows that Mg^2+^ favors 3SL over 2SL. However, such a small ΔΔG (several *k*_B_*T*) may not be sufficient to cause the 2SL
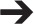
SL conformation switch required for the kissing interaction between 5BSL3.2 and 3′X. Other interactions, such as H-bond and base stacking, could play a more dominant role in stabilizing the 3SL fold. Similarly, for 5BSL3.2, as shown in Figure 1D, the ΔΔG between the kissing and non-kissing conformers (Figure 1C) indicates that Mg^2+^ favors the kissing conformation over the non-kissing conformation, suggesting that Mg^2+^ may induce the conformational switch for 5BSL3.2 from the non-kissing to the kissing structure to accommodate the base pairing with 3′X. The results may explain the NMR data that suggest the consolidation of the upper helix stem in the kissing structure as more Mg^2+^ ions are added to the solution.

The PCTBI predicted ΔG_ion_, the ion-induced electrostatic free energy, for the different 3D conformations provides insights about how ions “select” stable conformations (for a given 2D structure) for 5BSL3.2 and 3′X. For each 2D structure, the Vfold/MD approach produces thousands 3D conformations, which, according to the root-mean-square-deviation (RMSD), can be classified into 10, 10, and 7 clusters for the kissing conformation of 5BSL3.2, the 2SL conformation of 3′X, and the 3SL conformation of 3′X, respectively. The ΔG_ion_ profile for 3′X (see Figures 1E,F) shows that the lowest ΔG_ion_ structures are nearly unchanged for the concerned range of [Mg^2+^], while for (the kissing conformation of) 5BSL3.2, as shown in Figure 1G, an increase in [Mg^2+^] results in a switch of the lowest ΔG_ion_ structure from #9 (Mg^2+^-free solution; Figure 1H) to #10 (Figure 1I), indicating a stronger ion sensitivity of 5BSL3.2 than 3′X.

From the structure point of view, the kissing conformation of 5BSL3.2 has large hairpin and bulge loops (yellow and green in Figures 1H,I). Unlike structure #9, the loops in #10 bend further to the helix stems to form pockets, where the significant charge build-up captures more Mg^2+^ ions (red spheres). With the increase in [Mg^2+^], the difference in the number of the bound Mg^2+^ ions between #9 and #10 becomes further enlarged.

### Ion effects for the 5BSL3.2:3′X kissing complex

**Figure 2:**
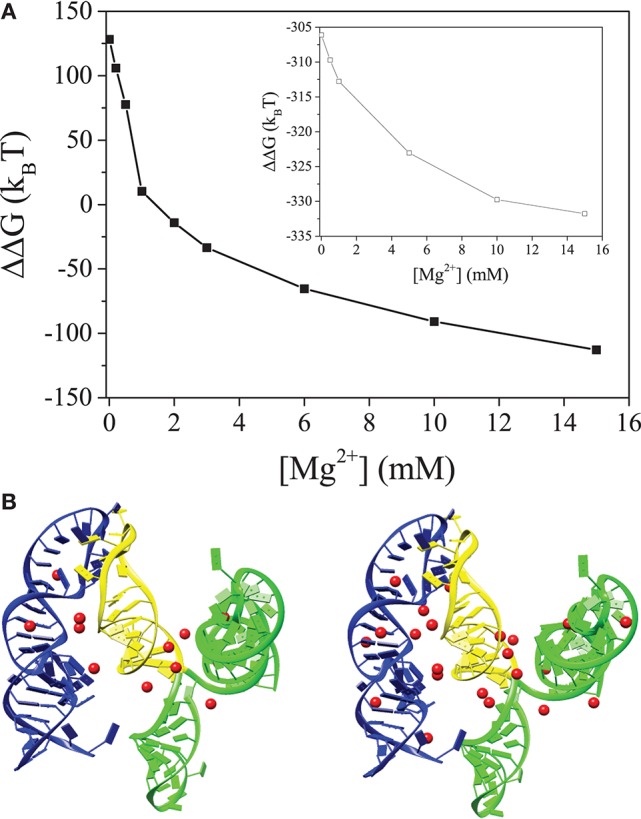
**(A)** The electrostatic free energy difference between the 5BSL3.2:3′X kissing complex and the unkissed structure. The inset show the free energy difference between the kissing and unkissed structures for 5BSL3.2:SL2 (yellow). **(B,C)** are the structures of the 5BSL3.2:3′X kissing complex with the predicted bound Mg^2+^ ions at [Mg^2+^] = 0.2 mM **(B)** and 2 mM **(C)**, respectively. 5BSL3.2 is labeled by blue and 3′X is labeled by green (SL1 and SL3) and yellow (SL2), respectively. Mg^2+^ ions are shown as red spheres. All the solutions contain 150 mM KCl buffer.

NMR studies suggest that 3′X adopts the 2SL conformation (undocked) in the absence of 5BSL3.2, and it remains unclear how the conformational switch to the docked conformation occurs in the presence of 5BSL3.2 and Mg^2+^ ions. The electrostatic free energy difference ΔΔG = ΔG_docked_-ΔG_undocked_ between the docked and undocked states for 5BSL3.2:3′X, as shown in Figure 2A, indicates a significant Mg^2+^-induced stabilization of the docked state. At dilute [Mg^2+^], ΔΔG >0 because the strong Coulomb repulsion between the negatively charged backbones on 5BSL3.2 and 3′X, preventing them from docking. For higher [Mg^2+^], ΔΔG <0 because as shown in Figures 2B,C, more Mg^2+^ ions would bind to the interface region between 5BSL3.2 and 3′X to reduce the electrostatic repulsion, promoting the 5BSL3.2:3′X docking.

Due to the higher charge density, the ion effects for the kissing complex (see Figure 2A) are much more pronounced than undocked 5BSL3.2 or 3′X (see Figure 1D). Although metal ions alone may not be sufficient to cause the conformational switch for 3′X, the higher concentration of ions can significantly enhance the probability for the close encounter and reaction (base pairing) between 5BSL3.2 and 3′X, which, in the meantime, can stabilize the 3SL form of 3′X. The presence of 5BSL3.2 makes the above ion-induced effects much more pronounced. Moreover, if we delete SL1 and SL3 (labeled by green in Figure 2) and keep only SL2 (marked yellow) in the 3SL fold for 3′X, the docked state is stabilized (ΔΔG < < 0; See the inset in Figure 2A) even in a Mg^2+^-free 150 mM KCl solution, indicating the dominant role of SL1 and SL3 in the repulsion between 5BSL3.2 and 3′X. In summary, Mg^2+^ promotes the long-range kissing interactions in two layers: (i) it stabilizes the 5BSL3.2 into the kissing-loop residue exposed conformation, and (ii) it reduces the electrostatic repulsion and stabilizes long-range intra-molecular base pairings.

## Discussion and conclusion

The combined theory-experiment studies described above demonstrate a general approach for the prediction and data interpretation for RNA structure and functions. The results for the metal ion effects are biologically significant. For the HCV system, the undocked and docked kissing-loop conformations are likely associated with distinct replications steps. Blocking the 5BSL3.2:3′X kissing-loop interaction suppressed viral protein translation without affecting RNA synthesis (Tuplin et al., 2015). Host protein EWSR1 was discovered to promote HCV RNA synthesis by preferentially interacting with 5BSL3.2 in the absence of 3′X (Oakland et al., 2013). Thus, the 3′-end of gRNA may fine-tune various functions utilizing a Mg^2+^ modulated structural equilibrium. The Mg^2+^ effect on the HCV gRNA is not limited to the 3′-end. It was recently reported that the IRES switched from an elongated structure to a closed comma-shaped structure when the Mg^2+^ concentration increased, and the structural change might be related to the observed different translation activities (Swaminathan, 2003; Romani, 2011; Garcia-Sacristan et al., 2015). Thus, the Mg^2+^-dependent structural equilibria in the HCV gRNA are likely an adaptive property to the intracellular Mg^2+^ concentration in liver cells for viral replication.

## Author contributions

All authors listed have made a substantial, direct and intellectual contribution to the work, and approved it for publication.

### Conflict of interest statement

The authors declare that the research was conducted in the absence of any commercial or financial relationships that could be construed as a potential conflict of interest. The reviewer FD and handling Editor declared their shared affiliation.
